# Migration policies versus public health – the ethics of Covid-19 related movement restrictions for asylum seekers in reception centers in Greece in 2020

**DOI:** 10.12688/wellcomeopenres.20547.1

**Published:** 2024-03-01

**Authors:** George Makris

**Affiliations:** 1 Department of Neurology, Venizeleio General Hospital, Heraklion, Crete, 71409, Greece

**Keywords:** Public health, ethics, movement restrictions, migration, infectious diseases

## Abstract

**Background:**

The emergency context of the Covid-19 pandemic necessitated the use of national and international public health measures of unprecedented scale to minimize mortality and morbidity, often in conflict with other principles and rights, such as the autonomy of individuals. Concerns have been voiced that for populations facing precarity, such as migrants, a disproportionate and unfair application of restrictive measures, deficient application of protective measures, and even enforcement of restrictive migration policies under the pretext of the pandemic has occurred.

**Methods:**

Various principles have been proposed as moral foundations of public health interventions. The author used two public health ethics frameworks to examine the acceptability of movement restrictions on asylum seekers residing in refugee camps in Greece from March 2020 to October 2020.

**Results:**

Most of the principles described in the frameworks for the ethical application of movement restrictions were not adhered to. Main concerns include that, measures were prolonged despite lack of evidence about their effectiveness to reduce morbidity and mortality, while posing severe and disproportionate burdens for this population.

**Conclusions:**

An ethically acceptable public health response to Covid-19 is incompatible with certain living conditions of refugees, asylum seekers, and migrants. The question of whether and if so the extent to which the discipline of public health inherently has the role of rectifying existing injustices and social inequalities when these can be convincingly related to health outcomes, is central to the design of public health interventions for these populations. The answer can exemplify the need to address moral and political determinants of health. It is essential for public health professionals to be aware of the moral theorizations that underpin their work, so as to ensure that their policies are aligned with those and to contribute to the debate that shapes these determinants.

## Plain Language Summary

In recent years, thousands of individuals have sought asylum in Greece. They are initially geographically restricted and accommodated in reception centers. These centers, or camps, have attracted international criticism due to undignified and harmful living conditions. In 2020, in the beginning of the CoViD-19 pandemic, the Greek government, imposed strict movement restrictions for camp residents in the name of public health. In this study, the author attempts to examine if the public health measures imposed on asylum seekers residing in reception centers in Greece in 2020 were ethically sound, drawing information from scientific articles, legislative acts and reports from a range of institutions.

Public health measures imposed during the pandemic have challenged individual liberties with the aim to protect human lives. However, for vulnerable populations, such as asylum seekers, it is uncertain if certain measures did more good than harm and if they were used to their best interest. Public health interventions are based on trust and should adhere to ethical principles.

Analysis of available evidence using ethics tools seems to be unable to justify the implemented CoViD-19 response policy for asylum seekers in reception centers. Further research, as well as political will, are needed to determine if and how ethical public health policies for migrants are feasible on a national and international level.

## Introduction

Migration is an ancient phenomenon, but it is now increasingly recognized as a core determinant of health ^1^. In 2020, 281 million people (or 3.6 % of the world population) were living in a country different from the one in which they were born, with many more displaced within their own country ^2^. Causes of migration include labor migration, forced migration (that can be due to conflict, persecution, disaster or other reasons), human trafficking, and other forms of modern slavery and climate change related migration.

Migration is historically linked to public health challenges with a prominent ethical dimension. Known ethical challenges of migration health include policies such as mass mandatory screening for infectious diseases upon arrival and entry restrictions and deportation based on medical conditions such as HIV infection, leprosy, and hepatitis C among others. The notion that migrants are carriers of disease is the most powerful myth related to migration and health originating from ancient times, but recent evidence does not support a high risk of transmission to host populations, especially when health care and surveillance services in the destination country are inclusive ^1, 3^

States carry moral and legal obligations to guarantee the right to the highest attainable standard of health, regardless of location and migration status according to multiple human rights norms, arguably containing inclusion in public health policies and preventive care ^4^. Nonetheless, governments often enforce exclusionary policies, invoking other concerns such as national security. Indeed, in our times there is an inherent tension between health and migration policy goals, with the former usually aiming for inclusion and the latter for exclusion.

The emergency context of the Covid-19 pandemic necessitated the use of national and international public health measures of unprecedented scale in order to minimize mortality and morbidity and allegedly to protect national security ^5^, often in conflict with other principles and rights, such as autonomy. These emergency measures, including international travel controls extended to border closures and internal movement restrictions, that were declared on a global scale due to the Covid-19 pandemic had a profound impact on the mobility of migrants. These measures led to migrants’ (including refugees and asylum seekers) inability to flee from their countries of origin, face increased barriers to enter transit or destination countries, or be stranded in places with overcrowded and poor living conditions in transit or destination countries without access to health care and protection. Thus, for many countries, the state of emergency brought by the pandemic took precedence over the central international norms regulating migration, such as the principle of non-refoulement for refugees and asylum seekers, as evidenced by border closures ^2^.

Additionally, concerns have been voiced that in populations having precarious legal status, such as migrants, refugees ,and asylum seekers, there has been a disproportionate and unfair application of restrictive measures ^6, 7^, as well as a deficient application of protective measures. Perhaps even more worryingly, some policies have been seen as instrumentalizing public health to achieve migration policy targets ^6, 8^. In line with recognizing the importance of the moral and political determinants of health ^9^, examining the evidence and shedding light on the effectiveness and justice of implemented public health policies can be seen as a moral obligation of (public) health professionals.

In the beginning of 2020 and at the start of the pandemic, 56 000 asylum seekers and refugees were estimated to reside in Reception and Identification Centers and Temporary Accommodation Sites in Greece ^7^. Mass quarantine and other restrictive measures were applied for longer periods and to a greater degree to encamped asylum seekers than for the general population with the stated objective of reducing transmission ^10^. On the other hand, substandard living conditions, overcrowding inside these camps, and challenges in accessing health care raised questions about the possibility of increased infection and other health risks inside these structures. Consequently the overall effectiveness of the said restrictive measures for the refugee and general population, as well as its ethical acceptability can be put into question.

The ethical acceptability of public health interventions has been theorized as justifiable on a number of conditions, such as overall benefit, collective action and efficiency, fairness in the distribution of burdens, prevention of harm, paternalism, least restrictive means, transparency, and various other related (and sometimes overlapping) moral concepts ^11, 12^. The first objective of this study is to examine the ethical acceptability/soundness of movement restrictions on migrants living in camps (mainly refugees and asylum seekers) in Greece from March 2020 to October 2020 in comparison to the measures applying to the general population. The second objective is to discuss the relevant ethical considerations that should be taken into account by policy makers when designing public health interventions for these vulnerable populations.

## Methods

### Study definitions

Different legal categories of migrants are used in this article, such as asylum seekers and refugees that mainly reflect the need for states to classify migration. It is important to highlight that these categories do not necessarily reflect individual needs or vulnerabilities.

Reception Identification Centers (RICs) or “hotspots” are defined as the six refugee centers at migration entry points in Greece in 2020 (five in the eastern Aegean islands and on the land border with Turkey), designed to process asylum applications. The hotspots “aim to improve coordination of the EU agencies' and national authorities' efforts at the external borders of the EU, in the initial reception, identification, registration and fingerprinting of asylum-seekers and migrants” ^13^.

Temporary accommodation sites are defined as the 32 camps in mainland Greece in 2020, designed to “offer temporary accommodation to third-country nationals or stateless individuals who have applied for international protection within the territory of Greece” ^14^.

### Ethical frameworks for the evaluation of movement restrictions

Two ethical frameworks for public health interventions were used in an examination of the ethical soundness of movement restrictions implemented for residents of reception and identification centers and refugee mainland camps.

Kass’ ethics framework for public health employs six open-ended questions for the ethics analysis of any public health program (not specific to infectious diseases) ^15^. It is articulated in the context of the lack of a public health code of ethics, in opposition to a growing body of scientific literature on medical ethics, and is described as “a code to preserve the negative rights of citizens to noninterference” as well as a code to emphasize the affirmative obligations of public health and to reduce certain social inequities.

On the other hand, the ethics-driven policy framework for implementation of movement restrictions in pandemics proposed by Zadey *et al.* uses 11 unique but related ethical principles (harm, justifiability, proportionality, least restrictive means, utility efficiency, reciprocity, transparency, relevance, equity, accountability, and cost and feasibility) that can then be processed to answerable questions in order to generate 34 policy indicators ^16^. This process is equivalent to the “specification” of these general principles in the context of Covid-19 related movement restrictions. These ethical principles were derived from literature preceding the Covid-19 pandemic on movement restrictions for infectious disease outbreaks and other biohazards, such as bioterrorism threats, the 2003 SARS epidemic, the influenza pandemic, the Ebola outbreak response, and WHO guidance for managing ethical issues in infectious disease outbreaks. Here, we are attempting to use this tool additionally for a specific population group, despite its being designed for application at the national level. Following the authors’ example, a principle is considered as adhered to if at least half of the indicators are affirmative.

Many of the resources on the ethical dimension of infectious diseases outbreak and response draw upon the Siracusa principles, an established framework for evaluating the appropriateness of limiting certain fundamental human rights in emergency situations, that state that “any limitation of, or derogation from, rights obligations must be lawful, pursue a legitimate aim, be strictly necessary and proportionate, be non-discriminatory, of limited duration and subject to review”.

### Data sources

Secondary data about the movement restrictions in Greece in 2020 and the assessment of their impact on the health of the general population and refugees, asylum seekers, and migrants was collected from peer-reviewed original research journal articles archived in online databases, national and regional epidemiological surveillance reports, legislation from the Greek government and reports from EU bodies and non-governmental organizations. Detailed information about the specific sources of the data used in the application of the frameworks is provided in the data availability statement.

## Results

### Application of the ethics framework proposed by Kass

The ethics framework for public health proposed by Kass was applied to evidence regarding the movement restrictions for refugees, asylum seekers, and migrants residing in reception centers in Greece from March to October 2020 in order to consider the ethical implications of this intervention.

i. What are the public health goals of the proposed program?
  The public health goal of the movement restriction orders was to “take measures against the presentation of cases and the spread of COVID-19 in RICs” taking into consideration “the necessity of measures to protect public health, in order to prevent the spread of Covid-19 in crowded settings such as the RICs” as stated in the government’s ministerial circular of 20/03/2020 ^17^.
  Arguably, the ultimate aim of this intervention is the reduction of morbidity and mortality due to Covid-19. It is not explicitly stated for which population the benefit is expected but presumably it refers to residents and workers of the reception centers as well as the general population.
ii. How effective is the program in achieving its stated goals?
  At the time of the application of the first measures in March 2020, there was a lack of available scientific evidence for the effectiveness of different public health interventions for the reduction of morbidity and mortality due to Covid-19.
  As early as June 2020 the European Center for Disease Prevention and Control (ECDC) issued guidance for infection prevention and control reporting that “there is no evidence that quarantining whole camps effectively limits transmission of SARS-CoV-2 in settings of reception and detention, or provides any additional protective effects for the general population, outside those that could be achieved by conventional containment and protection measures” ^18^.
  Evidence produced after the implementation demonstrated that during the first epidemic wave (spring of 2020) the risk of infection was almost 28 times higher in refugees and asylum seekers residing in the 32 RSs on the Greek mainland compared to the general population (IPR: 27.66; 95% CI: 24.14-31.6) ^7^.
  Nonetheless, there are serious challenges in determining accurately the effectiveness of the program since Greece lacked a testing and contact tracing strategy for residents of reception centers and outbreaks during the period of the first wave were discovered by incidental testing of severe cases in hospital settings. Additionally, epidemiological surveillance systems were not in place for these settings. Consequently, significant underreporting of cases can be hypothesized, while there is a complete lack of data availability regarding clinical outcomes such as hospitalizations or deaths for migrants, refugees, and asylum seekers ^7^.
iii. What are the known or potential burdens of the program?
  Burdens to liberty and self-determination are the most transversal burdens of infectious disease–related movement restrictions. A relevant underlying consideration is the condition of the space within which people are restricted. Overcrowded settings ^19^, and inadequate access to water ^10^, sanitation ^19, 20^, and medical care ^7^ are associated with an increased risk of adverse health effects, including but not limited to infectious disease outbreaks.
  As part of these negative health effects, there is evidence from Greece and other settings that adverse conditions in refugee camps deteriorate the mental health of their inhabitants ^21^, including increased rates of psychiatric disorders ^22, 23^ even before Covid-19 related lockdown measures.
  Another consequence of the movement restrictions for asylum seekers was the freezing of asylum procedures, leaving people in a state of prolonged administrative detention and limbo (judicial appeals for rejected asylum seekers were also suspended) ^8^. The uncertainty about their future was compounded by the reduction of services for education, recreational activities, legal representation, access to essential items, and self-sustenance strategies ^10^.
  Finally yet importantly, the stricter and longer application of movement restrictions for migrants contributed to xenophobic rhetoric, intensifying the representation of migrants as carriers of disease ^24^.
iv. Can burdens be minimized? Are there alternative approaches?
  The most notable measure to minimize burdens was the evacuation of a minority of residents that were classified as having comorbidities from camps in April 2020 in an attempt to shield the most vulnerable ^25^.
  Burdens could be further minimized if the setting of reception centers would allow for the implementation of standard community measures such as physical distancing, hand and respiratory hygiene measures in a setting of decent living conditions, as well as equitable access to testing, treatment, and care for reception center residents ^26^.
  Notwithstanding and due to the said structural and administrative barriers, physical distancing and risk-containment measures could not be safely implemented, further measures to de-congest and evacuate residents were not implemented ^18^.
v. Is the program implemented fairly?
  A system of exceptions in the restriction of movement for specific essential functions was applied to the general population by sending an automated message, but this was not tailored to the situation of migrants at reception centers ^10^.
  A different provision was implemented for residents of RCs, allowing a limited number of “representatives of families or groups to move to nearby urban areas to cover essential needs”, only if no recent case of Covid-19 was reported from the camp population. This led to frequent complete mass quarantine of the camps enforced by the police ^27^.
  Contravening ECDC recommendations and despite the lack of data proving effectiveness, the movement restrictions for migrants were prolonged until 12.10.2020 ^28^. During the summer of 2020, the general population and tourists (of which approximately 7.4 million visited Greece in 2020) could move freely in a context of “low transmission” ^10^, indicating another instance of an unequal distribution of burdens, without any scientific or moral justification ^10^. Additionally mandatory quarantine upon arrival was implemented until 2023 despite this not implemented to any other foreigner entering Greece from abroad ^10^.
  Movement restrictions and complete “lockdowns” of migrant camps were envisioned as the equal application of the stay-at-home orders for the migrant population. However, for persons that are forced to live in overcrowded, unhygienic, and harmful conditions just based on their status as migrants, an unfair distribution of burden can be claimed for an uncertain health benefit (or even harm) for this community. This approach can be even further questioned due to a lack of a tailored testing strategy and adequate access to health services that would correspond to the increased risk of infection, in a fair and rational distribution model.
vi. How can the benefits and burdens of the program be fairly balanced?
  Despite the highly burdensome nature of the movement restrictions for migrants, public hearings or any kind of consultation with migrant communities were not undertaken in Greece. Additionally, the national Covid-19 helpline was not operative in most languages spoken by migrants. No government in the EU produced risk communications on disease prevention targeting people in refugee camps or informal settlements at that time ^29^.

### Application of the ethics framework proposed by Zadey *et al.*

A description of the main characteristics of the movement restrictions imposed on residents of reception centers from March to October 2020 is provided in the analysis of the first framework above. The author’s assessment based on the framework proposed by Zadey *et al.* is presented in Table 1 ^30^, in comparison with the performance of the indicators for measures that applied to the general population. According to this model, the principles of justifiability, proportionality, least restrictive means, reciprocity, utility-efficiency, transparency, equity, and relevance can be considered as not adhered to for the migrants, while more than half of the performance indicators had an affirmative answer for the general population of Greece.

**Table 1.** Assessment of the framework proposed by Zadey *et al.* regarding COVID-19 related movement restrictions in 2020 for the general population and encamped migrants.

Ethical Principle	Questions/considerations raised under the principle	Policy monitoring and evaluation indicators for response measures	Performance of indicator for general population	Performance of indicator for encamped migrants
**Harm and Necessity**	Is there measurable harm due to the disease outbreak?	Presence of scientific evidence indicating harm (e.g., human to human transmission, mortality)	Yes	Yes
		Decided metric for harm measurement (e.g., death count, case count)	Yes	Yes
**Justifiability**	Is there scientific evidence for the effectiveness of the restriction to prevent/ reduce harm?	Presence of prior peer-reviewed scientific publications on the effectiveness of restrictions	Yes	No
	Is the restriction being withdrawn when new evidence suggests that the original rationale is no longer applicable?	Successful historical precedent (any instance before)	Yes	No
	Is the appropriateness of the restriction being continuously re-evaluated as and when more evidence emerges and when the course of the outbreak changes (increase/decrease in cases)?	Presence of a dedicated response team for review of literature, adequate data collection, impact evaluation and situational monitoring to continuously determine the effectiveness of the restrictions	Yes	No
**Proportionality**	Is the restriction proportional to the potential harm?	Matching stringency of restrictions with the growth of cases and deaths in the epidemic	Yes	No
**Least restrictive means**	Is the least restrictive measure applied before other measures severely curbing individual and communal rights?	The number of steps between the least (travel bans) and the most restrictive (national lockdown) measures?	Stepwise approach	Lockdown as first step
	Are voluntary restrictions implemented before mandatory restrictions?	Whether sufficient time intervals are given for every restrictive step to show the maximum effect?	Yes	No
**Utility Efficiency**	Do the probable benefits of the restriction outweigh the probable risks?	Does the analysis of trade-off (e.g., cost–benefit analysis) between loss of livelihood and other losses against deaths averted and cases averted show net positive benefit?	Likely	Unlikely
**Reciprocity**	Is the government reimbursing the individuals for curtailing their rights and for the loss of income/ loss of livelihood due to restrictions?	Cost and population coverage	Yes	No
	Is the government placing relief mechanisms ensuring that the restricted individuals are not facing an undue burden?	Tax and loan payment concessions	Yes	Not applicable
	Is the government placing relief mechanisms ensuring that the restricted individuals are not facing an undue burden?	Postponing non-essential routine activities (e.g., examinations, sports events, etc.)	Yes	Yes
	Are the societal groups less affected by the restriction taking care of those affected gravely by them?	Anti-discriminatory mass media practices	Yes	No
		Presence of helplines to deal with mental health issues that may arise	Yes	No
		Surveys for awareness among people about avoiding discrimination	No	No
		Presence of grassroots ventures that help the impoverished groups	Yes	Yes
**Transparency**	Are the policy decisions regarding restrictions and their rationale being continuously informed to the public in comprehensible ways?	Presence of press conferences in local languages	Yes	No
		Frequency of COVID- 19 press conferences	Daily	Not in local languages
		Presence of outreach methods and materials that are easy to understand, in local languages, and widely distributed	Yes	No
		Presence of a public record of justification for the quarantine that is conveyed to lay people in local languages	Yes	No
**Relevance**	Are the restrictions being implemented with feedback from the community that is affected by them?	Presence of public opinion polls	Yes	No
		Presence of people’s representatives in the decision making process of dedicated response teams	No	No
**Equity**	Is there equitable distribution of the available resources for relief to the marginalized communities?	Presence of food and shelter security for the below poverty line and low-earning unorganised labour groups affected by the restrictions	No	Not applicable
	Is there a disproportionate burden of the restriction on vulnerable populations?	Presence of domestic violence helplines for women and children	Yes	No
		Availability of healthcare access for the chronically ill and elderly groups	Yes	Emergency access only
**Accountability**	Are there measures in place for individuals to express grievances and challenge the restrictions?	Presence of grievance redressal and feedback portals	No	No
	Can the decision-makers be held accountable in case of losses (economic/ health)?	Presence of public platforms to challenge the restrictions by speaking to authority figures	No	No
		Presence of laws that allow for a process of appeal	Yes	Yes, but no access
		Uninterrupted and autonomous working of the judicial system for fast-tracking the restriction-related appeals	No	No
		Mechanisms for demanding reparations in case of life and livelihood losses due to restriction	Yes	Yes, but no access
**Cost & Feasibility**	Are financial and other resources available to carry out a restriction?	Presence of laws that allow the implementation of restriction	Yes	Yes
	Does the country have legal and disciplinary systems in place to enforce the restriction?	Presence of a police force for restriction (e.g., confinement) implementation	Yes	Yes
	Are there enough resources to provide food, shelter, counselling to the community during the period of restriction?	Ability to create places of confinement for restricted (e.g., quarantined/isolated) individuals	Yes	Partially

## Discussion

### Main findings

We have applied two distinct frameworks to evaluate the ethical soundness of the movement restrictions imposed upon asylum seekers, refugees, and migrants living in reception centers between March and October 2020.

According to the application of the first framework, this intervention failed to achieve its goals for reduction in morbidity and mortality due to Covid-19 for this population and was prolonged despite lack of evidence about its effectiveness. It posed severe burdens for the physical and mental health of migrants, while increasing stigmatization against them in public discourse. Other public health interventions, such as inter-community protective measures (hygiene and physical distancing) could not be applied in the context of the camps and access to health care was reduced. The movement restrictions were stricter and longer than for the general population, without adequate justification or consultations with migrant communities. No helplines or other procedures for grievances were available. The alternative approach to evacuate the camps was not preferred. Thus, it can be claimed that most of the questions do not have satisfactory answers to consider the movement restrictions, as implemented in this context, ethically sound.

Using the author’s assessment of the second framework, only 2 out of 11 principles were respected for this population in comparison to 9 out of 11 for the general population of Greece, as evidenced by the performance of derived indicators. Specifically, the principles of justifiability, proportionality, least restrictive means, reciprocity, utility-efficiency, transparency, relevance, and equity were not adhered to vis a vis this public health intervention.

The lack of ethically acceptable policies for encamped migrants in Greece’s public health response in 2020 represents a critical flaw in the management of the first period of the pandemic.

### Strengths and limitations of the ethical frameworks

The ethical frameworks that were used have different methodologies but can be considered complementary. Kass’ framework represents a more qualitative and narrative approach that can encompass the particularities of public health interventions with open-ended questions, while the framework of Zadey *et al.* is more specialized and attempts a quantitative grading of the ethical acceptability of movement restrictions.

Ethical frameworks do not represent rigorous scientific tools, have inherent validity limitations and cannot aspire to unequivocally solve ethical dilemmas. Indeed, overlapping questions and ethical principles can be found in the tools that were used in this study. Childress *et al.* note, “the terrain of public health ethics includes a loose set of general moral considerations – clusters of moral concepts and norms that are variously called values, principles or rules – that are arguably relevant to public health” ^12^ to which different weighing can be “assigned”. Methodologies based on “mid-level” moral principles or “principlist” approaches have been both criticized and defended for not aspiring to the status of a universal moral theory that can prioritize between different moral elements ^31^.

### Policy and practice implications and conclusions

Despite the existence of different moral approaches and epistemological limitations, the use of ethical frameworks by public health policy makers and independent public health professionals could serve as an aid to analyze the issues at stake, safeguard the ethical soundness, or even advocate for the abolition of programs.

A central issue in the design of public health interventions for migrants is the possible structural tension with migration policy goals. In this light, Kass’ question, i.e. to which extent the discipline of public health inherently has the role of rectifying existing injustices and inequalities when these can be convincingly related to health outcomes, acquires a particular significance ^15^.

Indeed, health professionals’ call to states to improve the social and political determinants of migrants’ health and to carefully assess the need for punitive approaches has been reiterated many times in recent global health literature ^1, 9, 32, 33^ and can be founded upon multiple and heterogeneous moral theorizations. From a deontological perspective, it is unquestionable that the protection of the health of any human being has the legitimacy of a universal law. From a utilitarian perspective, the evidence of the benefits of migrants in high-income countries is mounting, in terms of positive economic effects and lack of harmful effects to the health of host populations ^1^. Additionally, the call for equitable inclusion of migrants in public health plans and responses can be viewed as enlightened self-interest in a global reality that continuously generates migration due to war, climate change, and structural inequality. Arguably, protecting migrants’ health is also consistent with the four key principles of biomedical ethics (beneficence, non-maleficence, respect for autonomy, justice) and other principles that have been applied to public health interventions, such as the principle of solidarity.

Public health ethics does not only limit its scope to the provision of moral justification of policies and programs, but also aims to critically examine their relationship to one another in a broader context and uncover gaps in relation to a more complete moral picture ^11^. Consequently, further research is needed on how to achieve equitable national and transnational health policies in a world where both migration and transborder health threats seem to be on the rise. What is required as a moral foundation of these policies is nothing less than moral reasoning regarding the equal valuation of lives of citizens and non-citizens on a global level and in the face of states of emergency, despite the reality of political antagonism and diversity of ethical norms.

In this case study and in the context of the Covid-19 pandemic, the evacuation of refugee camps with substandard living conditions, would exemplify a public health intervention that addresses both an urgent health threat and the underlying determinants of health, such as migration policies. Attempts to imitate response measures for the general population, without taking into account the special characteristics and living conditions of this population, can result in policies of questionable effectiveness and morality, which can hardly be categorized under the heading of “health policy”.

## Data Availability

No primary source data were used for this article. Key documents and their sources are presented in the table below.
