# Supplementary material for: Migration policies versus public health – the ethics of Covid-19 related movement restrictions for asylum seekers in reception centers in Greece in 2020
Source: Wellcome Open Res. 2024 Dec 11;9:115. Originally published 2024 Mar 1. [Version 3] doi: 10.12688/wellcomeopenres.20547.3 (PMC11409434; doi:10.12688/wellcomeopenres.20547.3)
Supplement: Peer review history

# Reviewer report: Reviewer response for version 3

**DOI:** 10.21956/wellcomeopenres.25933.r116198

**Published:** 2025-01-21

**Authors:** Ilker Kayi

**Affiliations:** 1 Department of Public Health, Koc University, İstanbul, Turkey

Thank you for the opportunity to review the article "Migration Policies versus Public Health – The Ethics of Covid-19 Related Movement Restrictions for Asylum Seekers in Reception Centers in Greece in 2020", which critically examines the ethical dimensions of pandemic measures applied to migrants. The study underscores the importance of addressing the structural tension between migration policies and public health ethics to ensure equitable and effective responses in future emergencies.

 Please find my comments below:

**Introduction:**

 The introduction contextualizes migration within a global framework, emphasizing its increasing prevalence and implications for public health. The emphasis on the tension between migration policies and public health goals serves as a useful tool for the subsequent ethical analysis. However,

1. The rationale of the use-case to focus only on Greece should be explained in detail. It would be good to read how the authors see the European migration landscape that would feed into these travel restrictions. 
2. This would allow also to set the scene for inherent political tensions regarding securitization of migration processes through justifications based on public health concerns. 
3. While the objectives focus only on travel bans or movement restrictions, the introduction can provide a brief information about the prior status of migrants in the pre-pandemic context. 
4. This raises the issue of what kind of restrictive measures were applied in Greece in general and how these were different in terms of applying it to different populations such as migrants.

**Methods:**

 The application of two ethical frameworks contributes to the structure of the article. While the methodology demonstrates an effort to use established frameworks for ethical analysis, there are several strengths and limitations that could be addressed to improve the robustness and clarity of the study. Please see my comments and recommendations below:

1. In parallel to my last comment under Instruction section, it is important to understand what measures are under scrutiny in the article with proper definitions. Do travel restrictions include mass quarantine interventions, because as the authors mention they have been applied for longer periods and to a greater degree? These measures are also restricting the liberty of movement, which makes it a subject of ethical evaluation.
2. Study design and definitions should be different sections and study design should be incorporated with ethical framework and data collection parts. 
3. Data collection part should be in the format of a systematic review steps. The author has already provided the search criteria however, types of documents, number of documents in each type and selection criteria and selection procedures should also be clear. If possible, a flow chart would be helpful to understand the selection process.  
4. The analytical framework of the study is unclear. It obviously demonstrates the use of ethical frameworks however how the answers given to the questions stated in the ethical frameworks are not described. Are they all based on author opinion? Are they based on relevant documents? If so, how the documents are used to extract the relevant information? 

**Results:**

 I believe the results section is a well written and it is really noteworthy to compare the local population and migrants with an emphasis on structural determinants. My comment on the results section should be read in line with the comments regarding methods section. 

1. While using two frameworks serves as an enriching approach, there needs to be a process to integrate the findings to provide a more cohesive narrative. 

**Discussion:**

 The discussion clearly emphasizes the ethical problems with policy implications and role of public health. My comments to improve the section are below:

1. The broader policy background and how it can be part of these ethical problems can be discussed. Examples can be contextualizing the position of Greece in adopting the European migration policy, discussion how policy tensions between public health interventions and border security were handled in Greece etc.
2. Different country examples could provide a broader understanding. 

Best regards.

Is the work clearly and accurately presented and does it cite the current literature?

Yes

If applicable, is the statistical analysis and its interpretation appropriate?

Not applicable

Are all the source data underlying the results available to ensure full reproducibility?

Yes

Is the study design appropriate and is the work technically sound?

Partly

Are the conclusions drawn adequately supported by the results?

Yes

Are sufficient details of methods and analysis provided to allow replication by others?

Partly

Reviewer Expertise:

Public Health, Migration, Public Mental Health, Research Ehics

I confirm that I have read this submission and believe that I have an appropriate level of expertise to confirm that it is of an acceptable scientific standard.

# Reviewer report: Reviewer response for version 3

**DOI:** 10.21956/wellcomeopenres.25933.r115445

**Published:** 2024-12-13

**Authors:** Rachel Fabi

**Affiliations:** 1 SUNY Upstate Medical University, Syracuse, USA

Thank you to the author for these responses. My concerns have been sufficiently addressed.

Is the work clearly and accurately presented and does it cite the current literature?

Yes

If applicable, is the statistical analysis and its interpretation appropriate?

Not applicable

Are all the source data underlying the results available to ensure full reproducibility?

No source data required

Is the study design appropriate and is the work technically sound?

Partly

Are the conclusions drawn adequately supported by the results?

Partly

Are sufficient details of methods and analysis provided to allow replication by others?

Yes

Reviewer Expertise:

public health ethics; immigration; health policy

I confirm that I have read this submission and believe that I have an appropriate level of expertise to confirm that it is of an acceptable scientific standard.

# Reviewer report: Reviewer response for version 2

**DOI:** 10.21956/wellcomeopenres.25128.r95779

**Published:** 2024-09-17

**Authors:** Rachel Fabi

**Affiliations:** 1 SUNY Upstate Medical University, Syracuse, USA

Thank you for the opportunity to review this manuscript. This paper examines movement restrictions that were placed on asylum seekers living in reception centers in Greece during the early days of the COVID-19 pandemic through two ethical frameworks. The author develops an interesting and useful case study that demonstrates how ethical frameworks might be used to think through public health ethics cases during public health emergencies. I believe this is a valuable addition to the literature on the ethics of public health policies in the context of a pandemic and a thoughtful consideration of the ways migrant rights are often unjustly abrogated for reasons that ostensibly relate to public health but are in fact grounded in xenophobia.

 The paper could be improved in several discrete ways that could improve the readability and rigor of the work presented. I will divide my recommendations by section.

**Title/Abstract:**

 As a minor note, the title of the paper doesn’t fully work. The migration policies are not being weighed against public health, so “versus” doesn’t make a lot of sense here. Perhaps “and” instead of “versus” might better capture what the manuscript seeks to analyze?

 I am also a bit confused by some of the text of the abstract. In particular, the “Conclusions” section is worded in a way that is unnecessarily complex, to the point that some meaning may be lost: “The question of whether and if so the extent to which the discipline of public health inherently has the role of rectifying existing injustices and social inequalities when these can be convincingly related to health outcomes, is central to the design of public health interventions for these populations. The answer can exemplify the need to address moral and political determinants of health.” – these sentences do not have a clear meaning.

 Finally, so much of the abstract is written in the passive voice that it is difficult to understand. “Concerns have been voiced,” “Various principles have been proposed,” “were not adhered to” – these points would all benefit from identification of an actor.

**Introduction:**

 The author makes several broad, unsupported, or otherwise controversial claims without sufficient evidence or qualification:

- “Migration is historically linked to public health challenges with a prominent ethical dimension.” What does this mean? This paragraph might also benefit from an additional citation illustrating general issues at the intersection of migration and public health, e.g. [Fabi, R. E. (2019). Public health in the context of Migration: Ethics Issues related to immigrants and refugees. The Oxford Handbook of Public Health Ethics, 2019, 244-256].  
- Additionally, the author claims that “states carry moral and legal obligations to guarantee the right to the highest attainable standard of health…”. While this is an admirable assertion, it is not an uncontroversial claim, and is rejected by many states, despite the UN’s best efforts. This claim could stand some qualification. The citation here (#4) is also formatted oddly in the references.

This section also suffers from an overreliance on the passive voice, e.g.: “there are documented occurrences where ethnic and racial groups have been marginalized and portrayed as health threats” – by whom?

 The author introduces the idea of “ethical acceptability” in this introduction section but does not define it until the methods section. If the aim of this paper is indeed to examine ethical acceptability, it would be helpful to have this concept more fully explored in the introduction. As written, the reader is left wondering “acceptable to whom?” (and, indeed, even with the definition in the methods section, this concept remains underdeveloped).

**Methods:**

 Because this paper presents a public health case study, it does seem odd to organize it into “Methods” and “Results” as though it were a research paper. I imagine this decision must be due to journal requirements?

 The Siracusa Principles require a citation.

 The text on page 6, from “Main passages about the regulation describing movement restrictions in RIC” through the following two paragraph of text, seem out of context or insufficiently integrated into the paper. Is the “main passages” text meant to be a heading? And if so, I believe it would improve readability if the text following that heading were introduced and contextualized.

 I also think this paper would benefit from a better explanation up front of why the author is using two ethical frameworks, rather than choosing one and digging deeper into the selected option. In the strengths and limitations section, the author notes that the Kass framework is more useful for qualitative and narrative analysis, while Zadey’s framework is specialized and enables the “quantitative grading of ethical acceptability of movement restrictions.” It would be helpful to clarify this choice in the Methods section.

 That said, I’m not sure I fully understand this choice! The Kass framework is not really meant to be used in this way, performing a retrospective analysis of a program or policy that no longer exists, but rather is meant for the prospective comparison of possible interventions. Zadey’s framework seems like the much more appropriate and obvious choice for this analysis, and yet the author spends most of the actual writing of this paper answering the questions of the Kass framework (not always appropriately, as discussed below), while the entire Zadey framework is relegated to a table without discussion.

**Results:**

 As mentioned above, it seems odd to refer to the analysis of a policy through a framework as “results,” but I believe this may be a limitation of the form.

 Steps 1-3 and 5 of the Kass framework are applied well and thoroughly, but Kass steps 4 and 6 are underdeveloped. Specifically, step 4 doesn’t really make sense as written because the Kass framework asks prospective questions, and the author contemplates hypothetical responses of ways to minimize burdens that were not, ultimately, employed. This is confusing. Additionally, step 6 is not well-explained. Kass asks whether the benefits and burdens can be fairly balanced, and what this is really asking is whether the procedures for adopting the policy were fair. The author does not sufficiently explain the procedures for implementation.

 The Zadey analysis is even more perplexing, however, in that the entire analysis is relegated to a table. It is entirely unclear how the author arrived at the answers to the questions for each ethical principle in the framework, because it is not explained. I believe this paper would be much stronger if, instead of spending time on the Kass framework, the author instead unpacked the answers to each of the Zadey framework’s questions, with support/citations (many of which could come from the current Kass section). Given the late stage of this review, however, I suspect this substantial of a requested revision is beyond the scope of what may be possible for the author. I do not believe that such a change is necessary for indexing, and I think the author’s points remain valid even with the current dual-framework approach, imperfect though it may be.

 I also wish the author had spent more time explaining why they included performance indicators for the general population AND the RICs, instead of focusing solely on the RICs. If this was intended to draw conclusions about the fairness of applying different standards to different populations, which I believe it was, it would be helpful to have that spelled out explicitly.

**Discussion:**

 The second paragraph under “policy and practice implications” is redundant with the last paragraph of the strengths and limitations section. The references to deontological perspectives and utilitarian perspectives are somewhat confusing and do not sufficiently tie back to the previous analysis, so I recommend omitting these sentences. The final phrase (“… which can hardly be categorized under the heading of ‘health policy’”) doesn’t really land and could be omitted.

 I hope these comments are received in the spirit in which they are intended – I believe this paper offers valuable insights and a real contribution to the public health ethics literature by demonstrating the application of public health ethics frameworks to a real ethical dilemma. The author is clearly passionate and knowledgeable about the policy under discussion, and I believe consideration of a few changes to the presentation of the case study and analysis will strengthen the value of that contribution.

Is the work clearly and accurately presented and does it cite the current literature?

Yes

If applicable, is the statistical analysis and its interpretation appropriate?

Not applicable

Are all the source data underlying the results available to ensure full reproducibility?

No source data required

Is the study design appropriate and is the work technically sound?

Partly

Are the conclusions drawn adequately supported by the results?

Partly

Are sufficient details of methods and analysis provided to allow replication by others?

Yes

Reviewer Expertise:

public health ethics; immigration; health policy

I confirm that I have read this submission and believe that I have an appropriate level of expertise to confirm that it is of an acceptable scientific standard, however I have significant reservations, as outlined above.

# Response

**Published:** 2024-12-06

**Authors:** George Makris

**Affiliations:** Neurology, Venizeleio General Hospital, Heraklion, Crete, Greece

Thank you for the opportunity to review this manuscript. This paper examines movement restrictions that were placed on asylum seekers living in reception centers in Greece during the early days of the COVID-19 pandemic through two ethical frameworks. The author develops an interesting and useful case study that demonstrates how ethical frameworks might be used to think through public health ethics cases during public health emergencies. I believe this is a valuable addition to the literature on the ethics of public health policies in the context of a pandemic and a thoughtful consideration of the ways migrant rights are often unjustly abrogated for reasons that ostensibly relate to public health but are in fact grounded in xenophobia.

 The paper could be improved in several discrete ways that could improve the readability and rigor of the work presented. I will divide my recommendations by section.

**Response:** I am very grateful for the close examination of my work and your recommendations on how to improve it.  

**Title/Abstract:**

 As a minor note, the title of the paper doesn’t fully work. The migration policies are not being weighed against public health, so “versus” doesn’t make a lot of sense here. Perhaps “and” instead of “versus” might better capture what the manuscript seeks to analyze? **Response: **The title builds on the conclusion of the paper that the net effect of the movement restrictions was probably harmful in terms of public health. In that sense and in this specific case, there is a tension (thus the “versus”) between the given migration policies that supported the movement restriction and containment of asylum seekers and what ought to be done in terms of an equitable public health approach to the pandemic, which would probably include among others taking steps to evacuate the reception centers.  

 I am also a bit confused by some of the text of the abstract. In particular, the “Conclusions” section is worded in a way that is unnecessarily complex, to the point that some meaning may be lost: “The question of whether and if so the extent to which the discipline of public health inherently has the role of rectifying existing injustices and social inequalities when these can be convincingly related to health outcomes, is central to the design of public health interventions for these populations. The answer can exemplify the need to address moral and political determinants of health.” – these sentences do not have a clear meaning.  

**Response: **Rephrasing for more clarity in the revised version.

 Finally, so much of the abstract is written in the passive voice that it is difficult to understand. “Concerns have been voiced,” “Various principles have been proposed,” “were not adhered to” – these points would all benefit from identification of an actor.  

**Response: **Rephrasing. Some more notes on the deliberate use of passive voice can be found below.

**Introduction:**

 The author makes several broad, unsupported, or otherwise controversial claims without sufficient evidence or qualification:

- “Migration is historically linked to public health challenges with a prominent ethical dimension.” What does this mean? This paragraph might also benefit from an additional citation illustrating general issues at the intersection of migration and public health, e.g. [Fabi, R. E. (2019). Public health in the context of Migration: Ethics Issues related to immigrants and refugees. The Oxford Handbook of Public Health Ethics, 2019, 244-256].  
**Response: **The meaning of that sentence is that from an epidemiological perspective, migrants and travellers, more generally, can introduce or re-introduce pathogens that are non-existent or rare in destination countries that can lead to epidemic outbreaks or other adverse health effects. That fact, often led to the enforcement of quarantines, mandatory testing or other exclusionary practices by authorities on destination countries. I consider these public health practices ethically challenging, since there were often performed against the will of the concerned individuals. I am adding two more citations in the revised article that further illustrate these points.
- Additionally, the author claims that “states carry moral and legal obligations to guarantee the right to the highest attainable standard of health…”. While this is an admirable assertion, it is not an uncontroversial claim, and is rejected by many states, despite the UN’s best efforts. This claim could stand some qualification. The citation here (#4) is also formatted oddly in the references. **Response: **I am specifying my assertion in the revised version and fixing the citation.
- ​​​​​This section also suffers from an overreliance on the passive voice, e.g.: “there are documented occurrences where ethnic and racial groups have been marginalized and portrayed as health threats” – by whom?
**Response: **The passive voice is a conscious choice on this section. I think it is common experience that there are many voices in media and politics that portray immigrants as a source of health (and other) threats. I could cite many examples of that. McKann et al. have specifically investigated the role of the media on shaping narratives about migrants ^1^. In an example from the Greek context,  the minister of civil protection Mr. Chrisochoidis claimed in 2012 that “illegal immigrants living in overcrowded apartments in Athens are an insult to public health and morality” (among other things) and mandated the issuance of health certificates for immigrants ^2^ (citation in Greek). This choice reflects my belief that an investigation on the range of these opinions, as well as an evaluation of their credibility is an important and extensive topic in itself, but beyond the scope of my article. Independently of the prevalence and origin of these opinions, it is reasonable that they can trigger an investigation in the public health benefits and ethical acceptability of restrictive measures applied to migrants.
- The author introduces the idea of “ethical acceptability” in this introduction section but does not define it until the methods section. If the aim of this paper is indeed to examine ethical acceptability, it would be helpful to have this concept more fully explored in the introduction. As written, the reader is left wondering “acceptable to whom?” (and, indeed, even with the definition in the methods section, this concept remains underdeveloped).
**Response: **Ethics, in its simplest, is the study of what is right/good or what ought to be done. I am using the term ethical acceptability, simply as another way of the product of balancing benefits and harms and saying after careful consideration if a program is “good enough” to implement or if its past implementation was the right thing to do. In the Methods section, I provide a more general definition of how one may argue if a programme is “good enough”. On the one hand, there are burdens and restrictions of rights, which have to be justified enough through proportional health benefits, to reach a “good enough” public health intervention.   Obviously, this process of weighting benefits and burdens could differ between different moral valuations and principles. Thus, in this view, there is space for moral pluralism and that can be reflected in the use of different frameworks that may use different principles in the realm of public health ethics and beyond. At the same time, writing an ethics paper reflects a belief that others (experts, citizens, communities, policy makers) might be persuaded by arguments and that we can reach common, if not universal, ground on what ethically acceptable public health programmes are. As in many academic endeavors, the subject who is presupposed to consider these arguments is not any specific person or social group. Rather it is something like the Rawlsian subject, which has tries to have no subjective identifications or personal interests, tries to think under a “veil of ignorance” and reasons in favor of the common good. Some of the clarifications above were added to the relevant parts of the Introduction and Methods sections
- **Methods:** Because this paper presents a public health case study, it does seem odd to organize it into “Methods” and “Results” as though it were a research paper. I imagine this decision must be due to journal requirements?
**Response: **The IMRAD format is suggested but not required from the journal. I thought that the Methods section could facilitate the description and reproducibility of the research and the Results section could help in separating the application of the tools/frameworks from their interpretation in the Discussion section.
  The Siracusa Principles require a citation. Added, thank you
- The text on page 6, from “Main passages about the regulation describing movement restrictions in RIC” through the following two paragraph of text, seem out of context or insufficiently integrated into the paper. Is the “main passages” text meant to be a heading? And if so, I believe it would improve readability if the text following that heading were introduced and contextualized.
**Response: **Revised for readability.
- I also think this paper would benefit from a better explanation up front of why the author is using two ethical frameworks, rather than choosing one and digging deeper into the selected option. In the strengths and limitations section, the author notes that the Kass framework is more useful for qualitative and narrative analysis, while Zadey’s framework is specialized and enables the “quantitative grading of ethical acceptability of movement restrictions.” It would be helpful to clarify this choice in the Methods section. .
  That said, I’m not sure I fully understand this choice! The Kass framework is not really meant to be used in this way, performing a retrospective analysis of a program or policy that no longer exists, but rather is meant for the prospective comparison of possible interventions. Zadey’s framework seems like the much more appropriate and obvious choice for this analysis, and yet the author spends most of the actual writing of this paper answering the questions of the Kass framework (not always appropriately, as discussed below), while the entire Zadey framework is relegated to a table without discussion.  
**Response: **Explained in the Methods Section. I hoped that by using two different frameworks, in terms of methodology and focus, I could strengthen the validity of my conclusion. On your second point, while I am aware that the Kass framework is meant for prospective analysis, I am not sure I can find a convincing reason that it cannot be used also retrospectively. Additionally, an ambiguity in the temporal specification of the questions exists in the original paper by Kass. For example, question 5 (“Is the program implemented fairly?”) implies a program, which is already under implementation and not a possible intervention in the future ^3^. Regarding the development of the second framework, in the revised version I have chosen to expand also the indicators in text and supporting the answers with citations, while keeping the table to contrast with the general population.
- **Results:**
  As mentioned above, it seems odd to refer to the analysis of a policy through a framework as “results,” but I believe this may be a limitation of the form.
  Steps 1-3 and 5 of the Kass framework are applied well and thoroughly, but Kass steps 4 and 6 are underdeveloped. Specifically, step 4 doesn’t really make sense as written because the Kass framework asks prospective questions, and the author contemplates hypothetical responses of ways to minimize burdens that were not, ultimately, employed. This is confusing. Additionally, step 6 is not well-explained. Kass asks whether the benefits and burdens can be fairly balanced, and what this is really asking is whether the procedures for adopting the policy were fair. The author does not sufficiently explain the procedures for implementation.
  The Zadey analysis is even more perplexing, however, in that the entire analysis is relegated to a table. It is entirely unclear how the author arrived at the answers to the questions for each ethical principle in the framework, because it is not explained. I believe this paper would be much stronger if, instead of spending time on the Kass framework, the author instead unpacked the answers to each of the Zadey framework’s questions, with support/citations (many of which could come from the current Kass section). Given the late stage of this review, however, I suspect this substantial of a requested revision is beyond the scope of what may be possible for the author. I do not believe that such a change is necessary for indexing, and I think the author’s points remain valid even with the current dual-framework approach, imperfect though it may be.
**Response: **Answers to questions 4 and 6 of Kass’ framework are further developed in the revised text. Results from the second framework are further developed also in text.
- I also wish the author had spent more time explaining why they included performance indicators for the general population AND the RICs, instead of focusing solely on the RICs. If this was intended to draw conclusions about the fairness of applying different standards to different populations, which I believe it was, it would be helpful to have that spelled out explicitly.
**Response: **Yes, it was to illustrate the application of dual standards. This clarification has been added in the revised version.
- **Discussion:**
  The second paragraph under “policy and practice implications” is redundant with the last paragraph of the strengths and limitations section. The references to deontological perspectives and utilitarian perspectives are somewhat confusing and do not sufficiently tie back to the previous analysis, so I recommend omitting these sentences. The final phrase (“… which can hardly be categorized under the heading of ‘health policy’”) doesn’t really land and could be omitted. **Response: **Revised to eliminate redundancies. I think the part with the deontological and utilitarian perspectives further elaborates how different moral theories can reach similar conclusions and support a cosmopolitan view of migration health. Last sentence revised.
  I hope these comments are received in the spirit idn which they are intended – I believe this paper offers valuable insights and a real contribution to the public health ethics literature by demonstrating the application of public health ethics frameworks to a real ethical dilemma. The author is clearly passionate and knowledgeable about the policy under discussion, and I believe consideration of a few changes to the presentation of the case study and analysis will strengthen the value of that contribution.        
**References ** 
  1. International Organization for Migration. *The Role of Media Narratives in Shaping Public Opinion Toward Refugees: A Comparative Analysis*. (United Nations, 2023). doi:10.18356/9789292685546.
  2. Λοβέρδος - Χρυσοχοΐδης: Υποχρεωτικό πιστοποιητικό υγείας για μετανάστες. *ΤΟ ΒΗΜΑ* https://www.tovima.gr/2012/04/01/society/loberdos-xrysoxoidis-ypoxrewtiko-pistopoiitiko-ygeias-gia-metanastes/ (2012).
  3. Kass, N. E. An Ethics Framework for Public Health. *Am J Public Health* **91**, 1776–1782 (2001).

# Reviewer report: Reviewer response for version 2

**DOI:** 10.21956/wellcomeopenres.25128.r90187

**Published:** 2024-09-13

**Authors:** Theodoros Fouskas

**Affiliations:** 1 Department of Public Health Policy, School of Public Health, University of West Attica, Athens, Greece

The author has sufficiently addressed the reviewers' recommendations in the updated text. Therefore, I have no further comments.

Is the work clearly and accurately presented and does it cite the current literature?

Yes

If applicable, is the statistical analysis and its interpretation appropriate?

Not applicable

Are all the source data underlying the results available to ensure full reproducibility?

No source data required

Is the study design appropriate and is the work technically sound?

No

Are the conclusions drawn adequately supported by the results?

Yes

Are sufficient details of methods and analysis provided to allow replication by others?

No

Reviewer Expertise:

NA

I confirm that I have read this submission and believe that I have an appropriate level of expertise to confirm that it is of an acceptable scientific standard.

# Reviewer report: Reviewer response for version 2

**DOI:** 10.21956/wellcomeopenres.25128.r90188

**Published:** 2024-08-13

**Authors:** Ietza Bojorquez

**Affiliations:** 1 Department of Population Studies, El Colegio de la Frontera Norte, Tijuana, Baja California, Mexico

I thank the author for addressing my previous observations.

**My remaining recommendation are:**

- I suggest adding the study aims and design to the Abstract.
- Provide a link or reference for “... the Covid-19 legislation database”
- I replicated the author’s search strategy in PubMed and obtained 76 results, some of which are clearly not relevant for this article. I suggest adding the inclusion/exclusion criteria to select scientific articles for analysis, and also the inclusion criteria for legislation articles and reports by NGOs, etc.
- I suggest the author reviews the phrase “Main passages about the regulation describing movement restrictions in RIC”. I understand it was added in response to a previous observation, but I think it’s difficult to understand for a reader who is not aware of the previous review. Maybe the authors could summarize the content of the two subsequent paragraphs, instead of copying them.

Is the work clearly and accurately presented and does it cite the current literature?

Partly

If applicable, is the statistical analysis and its interpretation appropriate?

Not applicable

Are all the source data underlying the results available to ensure full reproducibility?

Yes

Is the study design appropriate and is the work technically sound?

Partly

Are the conclusions drawn adequately supported by the results?

Partly

Are sufficient details of methods and analysis provided to allow replication by others?

No

Reviewer Expertise:

Migrant health, health policy, public health, epidemiology

I confirm that I have read this submission and believe that I have an appropriate level of expertise to confirm that it is of an acceptable scientific standard.

# Reviewer report: Reviewer response for version 1

**DOI:** 10.21956/wellcomeopenres.22745.r83856

**Published:** 2024-07-11

**Authors:** Theodoros Fouskas

**Affiliations:** 1 Department of Public Health Policy, School of Public Health, University of West Attica, Athens, Greece

The manuscript focuses on used two public health ethics frameworks to examine the acceptability of movement restrictions on asylum seekers residing in refugee camps in Greece from March 2020 to October 2020. This is a very pertinent issue and the author addresses a significant topic. The manuscript could be benefitted by a minor revision in terms of the articulation and presentation of the argument, connection between theory and research data. 

**Introduction**

 The section introduces the problem and it is informative regarding this particular issue in Greece, but is should be more focused on the Greek context. It could undergo a minor rewriting and include:

 a) Official data from the Hellenic Ministry of Migration and Asylum (about migrant/asylum seekers/refugees) ( https://migration.gov.gr/en/statistika/) and from the National Health Organization (EODY) ( https://eody.gov.gr/en/covid-19/) and expand this section.

 b) Information about the conditions, capacity at that time and more details on the facilities.

 c) A better connection with previous works on the topic. For example: 

1. Tsavdaroglou (2022)
2.  
https://migrationhealth.org/wp-content/uploads/2021/05/lancet-migration-situational-brief-greece-03-en.pdf 
3.  Kousis *et al. *(2022)
4. Sapounas *et al. *(2021)
5. Dimari (2021)
6. Mellou *et al.* (2022)
7. Elhabrouk (2020)
8. Fouskas *et al.* (2022)
9. Fouskas (2020)

d) Provide a point-by-point analysis of the measures implemented during the pandemic that impacted migrants/asylum seekers/refugees in the RICs and Accommodation Facilities in the mainland.

**Methods**

 Methods are appropriate and the fit between theoretical discussion and methodology is well formulated.

**Results**

 The assessment framework is well developed, and aids the author in his analysis, and in framing the findings. The results are of interest for practice, social and migration policy and society more generally. Here a better connection with previous research findings could be implemented. 

**Discussion**

 A  well-organized and compelling discussion section is provided as well.

**Conclusions**

 A short concluding section could be added.

Is the work clearly and accurately presented and does it cite the current literature?

Yes

If applicable, is the statistical analysis and its interpretation appropriate?

Not applicable

Are all the source data underlying the results available to ensure full reproducibility?

No source data required

Is the study design appropriate and is the work technically sound?

No

Are the conclusions drawn adequately supported by the results?

Yes

Are sufficient details of methods and analysis provided to allow replication by others?

No

Reviewer Expertise:

Sociology, Migration, Public Health

I confirm that I have read this submission and believe that I have an appropriate level of expertise to confirm that it is of an acceptable scientific standard, however I have significant reservations, as outlined above.

## References

[1] : Refugees’ caring and commoning practices against marginalisation under COVID‐19 in Greece. *Geographical Research* .2022;60(2) : 10.1111/1745-5871.12522 232-240 10.1111/1745-5871.12522

[2] : A COVID-19 outbreak among migrants in a hosting facility in Greece, April 2020. *J Infect Prev* .2022;23(5) : 10.1177/17571774221092568 235-238 10.1177/17571774221092568 36003137 PMC9389273

[3] : Challenging Mobilities in and to the EU during Times of Crises. *Springer Nature* .2022; 10.1007/978-3-031-11574-5 10.1007/978-3-031-11574-5

[4] : Impact of COVID-19 Pandemic on Refugees, Migrants and Asylum Seekers Living in Camps and Reception and Identification Centers in Greece in the Pre-Vaccination Period, February 2020 to May 2021. Summary of Epidemiological Findings. doi:10.21203/rs.3.rs-911182/v1 10.21203/rs.3.rs-911182/v1

[5] : The Emergence of a New Security Apparatus in Greece: The Securitization of the Refugee/Covid-19 Crisis Nexus. *PARTECIPAZIONE E CONFLITTO* .2021; 10.1285/i20356609v14i1p341 10.1285/i20356609v14i1p341

[6] : "Crisis Within A Crisis: A Comparative Analysis Of Covid-19’s Implications On Greece And Spain’s Migrant And Refugee Processing Policies".2020; Reference source

[7] : Racial and Ethnic Inequalities, Health Disparities and Racism in Times of COVID-19 Pandemic Populism in the EU: Unveiling Anti-Migrant Attitudes, Precarious Living Conditions and Barriers to Integration in Greece. *Societies* .2022; 10.3390/soc12060189 10.3390/soc12060189

[8] : Migrants, asylum seekers and refugees in Greece in the midst of the COVID-19 pandemic. *Comparative Cultural Studies - European and Latin American Perspectives,* .2020.; 10.13128/ccselap-12297 10.13128/ccselap-12297

# Response

**Published:** 2024-07-12

**Authors:** George Makris

**Affiliations:** Neurology, Venizeleio General Hospital, Heraklion, Crete, Greece

I am very grateful for your expert review and the provision of relevant resources. I am following your suggestions to enrich the Introduction paragraph by adding more information and links with other works, while trying at the same time to maintain relative brevity, which I believe helps with the readability of the article. At the same time, I am trying to provide all the necessary information to feed the frameworks but keeping a public health lens.

 Please note that I am including only peer-reviewed scientific articles. I have added a reference to the Ministry of Migration official statistics that support the claims about the number of asylum seekers in the island RICs. I don’t think that the National Public Health Organisations (EODY) website includes relevant information on cases at that period [March to October 2020], since there was no capacity to test people in refugee camps then. More information on the living conditions of people living in the camps is provided.

 It is noted that relevant measures are also described in the results section, under points of the first framework. Therefore, I am also adding some pertinent information there.

 Additionally the revised article contains a translation of the main regulations that affected the movement of camp residents under the Methods section.

 Conclusive remarks are provided in the last sub-section of the discussion titled “Policy and Practice implications and conclusions”

 Many thanks again.

# Reviewer report: Reviewer response for version 1

**DOI:** 10.21956/wellcomeopenres.22745.r81855

**Published:** 2024-05-21

**Authors:** Ietza Bojorquez

**Affiliations:** 1 Department of Population Studies, El Colegio de la Frontera Norte, Tijuana, Baja California, Mexico

In this article, the author applies two public health ethics frameworks to the regulations for restriction of movement affecting asylum seekers living in reception centers in Greece during March-October 2020, that were part of the country’s response to the COVID-19 pandemic. The author concludes that the restrictions did not conform to the standards for public health action as proposed in the two frameworks. This is an important work, which adds to the discussion of how the pandemic response affected migrants disproportionately, and what concrete steps could be taken to avoid repeating the same errors in the future. It also has the potential to inform broader discussions of public health ethics.

 In order to strengthen the article’s argument, I identify three major aspects that the author should address:

 First, the author needs to present the article’s objective(s) in a consistent and clear way throughout, and to define the concepts included in that(those) objective(s). In its present form,

 the Abstract presents the objective as “to examine the acceptability of movement restrictions on asylum seekers”. This immediately raises the questions of: what is meant by acceptability? and acceptability for whom? and creates the expectation of a study where migrants and other key informants are asked about their impressions regarding the movement restrictions. Later on, the author seems to use “acceptability” as shorthand for “ethical acceptability”, but still it’s not clear what is meant by this. Is it “ethical justification”, as in Faden & Sebaya (refer 1). Is this the same as “ethical soundness” (p. 5). In the Iast paragraph of the Introduction, the main objectives are listed as: “... to examine the ethical acceptability/soundness of movement restrictions on migrants living in camps...”, and “to discuss the relevant ethical considerations that should be taken into account by policy makers when designing public health interventions for these vulnerable populations...”. After reading the full article, I think the design, methods and results align reasonably well with the first objective, but the second one seems part of the background for the frameworks employed, rather than an actual aim. Also missing is a discussion of the relationship between the two proposed objectives. I suggest the author revises the wording of the objective(s), uses the same terms throughout the article, and defines them.

 Secondly, I think the author conflates two levels of analysis. From the description of the public health action being analysed (movement restriction), it seems this action was (in principle) developed for the protection of the whole population, and not just those in the reception centers. Therefore, the main analysis should consider if the action conformed with ethical principles at the level of the whole population (the results in Table 1 show it deed). The author makes a very interesting point that they “[attempt] to use [Zadey et al’s framework] additionally for a specific population group, *despite its being designed for application at the national level analysis*” (emphasis mine). What are the implications of doing this? I feel this contradiction is glossed over and not exploited sufficiently for analytical purposes.

 Maybe my previous comment is motivated by the limited description in the article of the public health action being analysed. To which action(s)/regulation(s) were the frameworks applied? Was it only “... the government’s ministerial circular of 20/03/2020”? or were other circulars included in the analysis? To clarify this, I suggest adding the following to the Methods section: 1) making explicit which regulations or other policy instruments (laws, circulars, protocols, etc.) were considered in the analysis; 2) a more detailed description of the measures included in those regulations/policy instruments (where all restrictions contained in a single document? what did they say exactly? did they change at different times? which ones applied only to the RICs and which ones were more general?); 3) a clarification of what were the two frameworks applied to: the written regulations, and/or their actual application?

 Third, the results presented are descriptive, followed by a reflection on the importance of applying ethical principles in public health work, especially with vulnerable populations. But there is no joint discussion of the results based in each of the two frameworks (e.g. are they complementary, contradictory?), nor of the usefulness of the moral reasoning behind those frameworks, or the particulars of the frameworks when applied to certain population groups. These are only a few lines of thought that came to my mind when reading this article, and I think they would enrich it and help to move forward the field of public health ethics and practice.

 Additionally, I have the following comments:

 INTRODUCTION

- “States carry moral and legal obligations to guarantee the right to the highest attainable standard of health...” In this paragraph, the author combines moral and legal obligations. Ideally, the two should align, but this is rarely the case (especially since moral obligations are not frequently the focus of policy/law making). I suggest distinguishing between the two.
- “Indeed, in our times there is an inherent tension between health and migration policy goals, with the former usually aiming for inclusion and the latter for exclusion” Has there been a time were this was not the case? I don’t think so, and even if this is the case, what’s the definition (time span) of “our times”?
- “The ethical acceptability of public health interventions has been theorized as justifiable on a number of conditions, such as overall benefit, collective action and efficiency, fairness in the distribution of burdens, prevention of harm, paternalism, least restrictive means, transparency, and various other related (and sometimes overlapping) moral concepts”. But the focus on this article is on the morality of a specific intervention (movement restriction), not on every possible public health intervention. I suggest highlighting the ethical/moral conflicts this specific intervention entails (as opposed to other public health measures), for example the tension between personal/individual liberties and public health goals (refer 2)

METHODS

- What’s the overall study design?
- The process for locating and selecting documents for the analysis is not clear. What was the searching protocol? What inclusion/exclusion criteria were employed? What happened if some sources contradicted others? It would be good to have a flow diagram of the document search and search results.
- What was the overall analytic strategy? What was the role in the analysis of the different types of documents (policy documents, reports, peer-reviewed literature, etc.)? Were the documents coded for analysis? Was there some sort of validation through multiple coders?
- The table under “data availability statement” lists “key documents”. Does it mean this were the more important documents, or these were the only ones analyzed?
- The second section of Methods is titled “Ethical frameworks for the evaluation of movement restrictions”. After describing the two that were employed, it mentions the Siracusa principles, making it seem these would be a third framework for the analysis.

Is the work clearly and accurately presented and does it cite the current literature?

Partly

If applicable, is the statistical analysis and its interpretation appropriate?

Not applicable

Are all the source data underlying the results available to ensure full reproducibility?

Yes

Is the study design appropriate and is the work technically sound?

Partly

Are the conclusions drawn adequately supported by the results?

Partly

Are sufficient details of methods and analysis provided to allow replication by others?

No

Reviewer Expertise:

Migrant health, health policy, public health, epidemiology

I confirm that I have read this submission and believe that I have an appropriate level of expertise to confirm that it is of an acceptable scientific standard, however I have significant reservations, as outlined above.

## References

[1] : Public Health Programs and Policies: Ethical Justifications.2019; 10.1093/oxfordhb/9780190245191.013.3 20-32 10.1093/oxfordhb/9780190245191.013.3

[2] : An ethics framework for public health. *Am J Public Health* .2001;91(11) : 10.2105/ajph.91.11.1776 1776-82 10.2105/ajph.91.11.1776 11684600 PMC1446875

# Response

**Published:** 2024-07-12

**Authors:** George Makris

**Affiliations:** Neurology, Venizeleio General Hospital, Heraklion, Crete, Greece

In this article, the author applies two public health ethics frameworks to the regulations for restriction of movement affecting asylum seekers living in reception centers in Greece during March-October 2020, that were part of the country’s response to the COVID-19 pandemic. The author concludes that the restrictions did not conform to the standards for public health action as proposed in the two frameworks. This is an important work, which adds to the discussion of how the pandemic response affected migrants disproportionately, and what concrete steps could be taken to avoid repeating the same errors in the future. It also has the potential to inform broader discussions of public health ethics.

**Response: I am very grateful for the detailed, incisive and clearly very informed review, which will definitely lead to the elucidation of many points in the article.**

 In order to strengthen the article’s argument, I identify three major aspects that the author should address:

 First, the author needs to present the article’s objective(s) in a consistent and clear way throughout, and to define the concepts included in that(those) objective(s). In its present form, the Abstract presents the objective as “to examine the acceptability of movement restrictions on asylum seekers”. This immediately raises the questions of: what is meant by acceptability? and acceptability for whom? and creates the expectation of a study where migrants and other key informants are asked about their impressions regarding the movement restrictions. Later on, the author seems to use “acceptability” as shorthand for “ethical acceptability”, but still it’s not clear what is meant by this. Is it “ethical justification”, as in Faden & Sebaya (refer 1). Is this the same as “ethical soundness” (p. 5). In the Iast paragraph of the Introduction, the main objectives are listed as: “... to examine the ethical acceptability/soundness of movement restrictions on migrants living in camps...”, and “to discuss the relevant ethical considerations that should be taken into account by policy makers when designing public health interventions for these vulnerable populations...”. After reading the full article, I think the design, methods and results align reasonably well with the first objective, but the second one seems part of the background for the frameworks employed, rather than an actual aim. Also missing is a discussion of the relationship between the two proposed objectives. I suggest the author revises the wording of the objective(s), uses the same terms throughout the article, and defines them.

**Below I will attempt respond point by point to the major comments:**

**Response: Regarding ethical acceptability/justifiability**

**Justifiability can be one of the principles of an ethically acceptable programme. I was using acceptability and soundness interchangeably. For clarity, I have changed all mentions to ethical acceptability when referring to the objectives of my study. As mentioned in Faden & Sebaya “Public health as a social institution draws its foundational moral legitimacy from the essential and direct role that health plays in human flourishing, whether that role is ultimately understood in terms of maximizing health or of promoting health in order to advance a broad conception of social justice.” Consequently, I use the concept of an ethically acceptable public health policy as one that must demonstrate adherence to the maximization of these social goods through a rational and coherent line of moral reasoning (such as principlism). Regarding the relationship between the two objectives, I believe it is analogous to the two aspects of every case study. The first is to elucidate and to reach a judgment about the specific case and the second is to try to consider the broader determinants of morbidity and try to address those. Specifically, the first objective is addressed by analysing the specific intervention of the state and the second by discussing general concerns (that are triggered by this case) about the interference of migration policies in public health interventions and how this can influence their moral character.**

 Secondly, I think the author conflates two levels of analysis. From the description of the public health action being analysed (movement restriction), it seems this action was (in principle) developed for the protection of the whole population, and not just those in the reception centers. Therefore, the main analysis should consider if the action conformed with ethical principles at the level of the whole population (the results in Table 1 show it deed). The author makes a very interesting point that they “[attempt] to use [Zadey et al’s framework] additionally for a specific population group, *despite its being designed for application at the national level analysis*” (emphasis mine). What are the implications of doing this? I feel this contradiction is glossed over and not exploited sufficiently for analytical purposes.

 Maybe my previous comment is motivated by the limited description in the article of the public health action being analysed. To which action(s)/regulation(s) were the frameworks applied? Was it only “... the government’s ministerial circular of 20/03/2020”? or were other circulars included in the analysis? To clarify this, I suggest adding the following to the Methods section: 1) making explicit which regulations or other policy instruments (laws, circulars, protocols, etc.) were considered in the analysis; 2) a more detailed description of the measures included in those regulations/policy instruments (where all restrictions contained in a single document? what did they say exactly? did they change at different times? which ones applied only to the RICs and which ones were more general?); 3) a clarification of what were the two frameworks applied to: the written regulations, and/or their actual application?

**Response:** **Different measures for camp residents and the general population**

**I apologize for this point not being clear in the article. In the introduction it is stated that: “Mass quarantine and other restrictive measures were applied for longer periods and to a greater degree to encamped asylum seekers than for the general population with the stated objective of reducing transmission”. In fact, two very different, and only to a small degree overlapping, sets of provisions and restrictions (in terms of severity and duration) applied to the general population and residents of reception centres. Therefore, it is justified to apply the frameworks just for the response in reception centers, as to answer the main research question. The comparison with the general population is then used to demonstrate the characteristics of the two approaches. The most important regulations used in the analysis were the ministerial circular of 22/03/2020 and the press briefing of 03/04/2023, which describes the activation of the “Agnodiki” plan on the 23/03/2022. A description of the phrasing of the measures will be added in the Methods section of the revised version. These measures were not changed from then on until October 2020, just extended multiple times. The frameworks were applied to the totality of the evidence collected, including the written regulations, as well as information about their application collected from the sources. For example the first point of the first framework regarding the public health goals is set out in the regulations but the fifth point regarding implementation is answered based on the data regarding implementation. Third, the results presented are descriptive, followed by a reflection on the importance of applying ethical principles in public health work, especially with vulnerable populations. But there is no joint discussion of the results based in each of the two frameworks (e.g. are they complementary, contradictory?), nor of the usefulness of the moral reasoning behind those frameworks, or the particulars of the frameworks when applied to certain population groups.**

**Response:** **Frameworks**

**As mentioned in the discussion, frameworks can be helpful, and even necessary, to ensure that relevant issues are considered by policy makers when a public health intervention is considered. About the two specific frameworks that I used, in the part of Strengths & Limitations, there is a mention of how they can be used as complementary tools, due to their different approaches. At the same time, the broader concern is that any answer based on a framework cannot be considered definitive or exhaustive to endorse an intervention since no framework can be expected to encompass all the nuances of every case. One relevant caveat, which I mention in the Strengths & Limitations paragraph, is that they cannot substitute for a universal moral theory (and do not necessarily aspire to). In this case, the frameworks may give a sufficient answer from a public health perspective, but definitely cannot account for the political and moral elements that shape the final product of the policy maker. In the discussion it is stated that “Public health ethics does not only limit its scope to the provision of moral justification of policies and programs, but also aims to critically examine their relationship to one another in a broader context and uncover gaps in relation to a more complete moral picture” Open question frameworks might be more suited for that purpose to expand on broader moral relations, such as the significance of the already disadvantaged position of asylum seekers in terms of social determinants of health. On the other hand, what I consider valuable in the more quantitative approach of the second framework is the ability to compare and contrast different interventions (between population groups and possibly even in different settings and different times). I am including elements of this response in the revised manuscript. These are only a few lines of thought that came to my mind when reading this article, and I think they would enrich it and help to move forward the field of public health ethics and practice.**

 Additionally, I have the following comments:

 INTRODUCTION

 “States carry moral and legal obligations to guarantee the right to the highest attainable standard of health...” In this paragraph, the author combines moral and legal obligations. Ideally, the two should align, but this is rarely the case (especially since moral obligations are not frequently the focus of policy/law making). I suggest distinguishing between the two. **Response:** **I am not suggesting that the two are identical. Rather that both can apply as moving forces for promoting migrant health.**

 “Indeed, in our times there is an inherent tension between health and migration policy goals, with the former usually aiming for inclusion and the latter for exclusion” Has there been a time were this was not the case? I don’t think so, and even if this is the case, what’s the definition (time span) of “our times”?

**Response:** **What I had in mind is the process of delegitimization of asylum and mistrusting asylum seekers as described by D. Fassin, which describes the declining rates of asylum recognition in Europe from the 1970s in contrast with the years following the Geneva Convention (Fassin, D. Policing Borders, Producing Boundaries. The Governmentality of Immigration in Dark Times. *Annu. Rev. Anthropol.* 40, 213–226 (2011)). Probably the tension is structurally inherent but may be intensified by such processes that influence the perception of asylum seekers. However, this discussion is probably out of the scope of this article, so I will remove the temporal specification from the sentence.**

 “The ethical acceptability of public health interventions has been theorized as justifiable on a number of conditions, such as overall benefit, collective action and efficiency, fairness in the distribution of burdens, prevention of harm, paternalism, least restrictive means, transparency, and various other related (and sometimes overlapping) moral concepts”. But the focus on this article is on the morality of a specific intervention (movement restriction), not on every possible public health intervention. I suggest highlighting the ethical/moral conflicts this specific intervention entails (as opposed to other public health measures), for example the tension between personal/individual liberties and public health goals (refer 2)

**Response: Indeed, I am trying to show that as for most burdensome public health interventions that restrict individual liberties, many justifications can be offered, which are all also pertinent in the specific case of movement restrictions.**

 METHODS

 What’s the overall study design?

**Response: Public Health Case Study**

 The process for locating and selecting documents for the analysis is not clear. What was the searching protocol? What inclusion/exclusion criteria were employed? What happened if some sources contradicted others? It would be good to have a flow diagram of the document search and search results.

**Response: The search strategy for this document review included searching the Covid-19 legislation database for all articles from the ministry of migration, through which 151 articles were identified and read for relevant information.  A search of the MEDLINE database was performed for relevant scientific articles using the search string ((Greece) AND (covid) AND (migrants OR migration OR asylum seekers OR refugees OR reception centres OR RIC)) Additionally, a targeted search for reports of EU bodies and NGOs working with migrants in Greece was performed. The strategy was complemented with snowball searching of all identified documents. Added the above in the Methods section.**

 What was the overall analytic strategy? What was the role in the analysis of the different types of documents (policy documents, reports, peer-reviewed literature, etc.)? Were the documents coded for analysis? Was there some sort of validation through multiple coders?

**Response: Drawing all the information that would be relevant for the application of the ethical frameworks. The documents were not coded for the analysis.**

 The table under “data availability statement” lists “key documents”. Does it mean this were the more important documents, or these were the only ones analyzed?

**Response: These were the most important documents for the application of the frameworks , the rest are listed as references.**

 The second section of Methods is titled “Ethical frameworks for the evaluation of movement restrictions”. After describing the two that were employed, it mentions the Siracusa principles, making it seem these would be a third framework for the analysis.

**Response: The Siracusa principles are mentioned as a historical and foundational document that influenced the content of the frameworks that were used in the analysis.**
